# Pepper EST database: comprehensive *in silico *tool for analyzing the chili pepper (*Capsicum annuum*) transcriptome

**DOI:** 10.1186/1471-2229-8-101

**Published:** 2008-10-09

**Authors:** Hyun-Jin Kim, Kwang-Hyun Baek, Seung-Won Lee, JungEun Kim, Bong-Woo Lee, Hye-Sun Cho, Woo Taek Kim, Doil Choi, Cheol-Goo Hur

**Affiliations:** 1 Omics Integration Research Center, KRIBB, 111 Gwahangno, Yuseong-gu, Daejeon 305-806, Korea; 2 School of Biotechnology, Yeungnam University, Gyeongsan, Gyeongbuk 712-749, Korea; 3 Plant Genome Research Center, KRIBB, 111 Gwahangno, Yuseong-gu, Daejeon 305-806, Korea; 4 Department of Biology, College of Life Science and Biotechnology, Yonsei University, Seoul 120-749, Korea; 5 Department of Plant Science, Seoul National University, Seoul 151-921, Korea

## Abstract

**Background:**

There is no dedicated database available for Expressed Sequence Tags (EST) of the chili pepper (*Capsicum annuum*), although the interest in a chili pepper EST database is increasing internationally due to the nutritional, economic, and pharmaceutical value of the plant. Recent advances in high-throughput sequencing of the ESTs of chili pepper cv. Bukang have produced hundreds of thousands of complementary DNA (cDNA) sequences. Therefore, a chili pepper EST database was designed and constructed to enable comprehensive analysis of chili pepper gene expression in response to biotic and abiotic stresses.

**Results:**

We built the Pepper EST database to mine the complexity of chili pepper ESTs. The database was built on 122,582 sequenced ESTs and 116,412 refined ESTs from 21 pepper EST libraries. The ESTs were clustered and assembled into virtual consensus cDNAs and the cDNAs were assigned to metabolic pathway, Gene Ontology (GO), and MIPS Functional Catalogue (FunCat). The Pepper EST database is designed to provide a workbench for (i) identifying unigenes in pepper plants, (ii) analyzing expression patterns in different developmental tissues and under conditions of stress, and (iii) comparing the ESTs with those of other members of the *Solanaceae *family. The Pepper EST database is freely available at .

**Conclusion:**

The Pepper EST database is expected to provide a high-quality resource, which will contribute to gaining a systemic understanding of plant diseases and facilitate genetics-based population studies. The database is also expected to contribute to analysis of gene synteny as part of the chili pepper sequencing project by mapping ESTs to the genome.

## Background

Pepper is a member of the family *Solanaceae*, which is one of the largest families in the plant kingdom and includes more than 3,000 species [1]. The *Solanaceae *family includes important crops, such as pepper, tomato, tobacco, potato, and eggplant and has been highly cultivated over the years for human nutrition and health. *Capsicum *species are consumed worldwide and are valued because of their unique color, pungency, and aroma. *Capsicum *peppers include *C. annuum*, *C. chinense*, *C. baccatum*, *C. frutescens*, and *C. pubescens *and are cultivated in different parts of the world. Of these, the varieties of the chili pepper plant species *C. annuum*, having a modest-sized diploid genome (2*n *= 24), are the most heavily consumed due to their nutritional value and spicy taste [2]. The chemical that is primarily responsible for the pungency of *C. annuum *has been identified as capsaicin [3], which elicits numerous biological effects and is the target of extensive investigation.

Expressed Sequence Tags (ESTs) are short subsequences derived from randomly isolated cDNAs [4]. With the advent of massive computational and biostatistical analysis, large-scale EST data sets can be efficiently analyzed to monitor gene expression [5-7]. ESTs in vegetable plants provide the opportunity to expand our knowledge of the genetic control of complex traits and the findings are applied in the agricultural industry to advance efforts to screen ecologically important phenotypes and reduce plant disease [8]. EST databases also provide comparative data for analyses of organisms that lack comparable genomic resources [9].

The development of automated high-throughput chili pepper EST sequencing projects in Korea has generated hundreds of thousands of EST sequences. Previous studies indicate that EST databases provide valid and reliable data for understanding gene expression and for gene mining [10]. Databases have been constructed for ESTs accumulated for tomato species to permit scoring of gene expression patterns *in silico*; these include the Tomato Stress EST (TSED), Micro-Tom (MiBASE) [11], and TomatEST databases [12]. Two pepper EST databases have been constructed, including the DFCI pepper gene index [13] and Pepper unigene at the sol genomics network [14]. Those databases were built on approximately 31,000 EST sequences, among the EST sequences, around 21,000 sequences were provided by our group; however, there has been a growing international need for a more comprehensive chili pepper EST database to enable extensive digital analysis of gene expression in pepper species because of the increasing interest in pepper's nutritional and pharmaceutical properties, as well as its spicy taste.

In this report, we present the Pepper EST database, a web-based database of of chili pepper plant ESTs. Pepper EST contains significantly more ESTs than existing databases (122,582 ESTs vs approximately 31,000 ESTs) and provides several advanced features, such as linking ESTs and their digital expression data. We constructed Pepper EST as a pipeline for comprehensive EST data analyses for investigations of expressed gene data. The database contains (i) raw sequence data; (ii) high-quality consensus sequences obtained from the assembly phase; (iii) tissue-specific ESTs; (iv) full-length cDNAs; (v) and functional annotation and assignment to metabolic pathways based on BLAST similarity searches. The unique feature of the Pepper EST database is the data set. ESTs were derived from cDNA sequences derived from different tissues of plants of a single chilli pepper variety, grown under constant growth conditions with exposure to a variety of stress agents.

## Results

**Figure 1:**
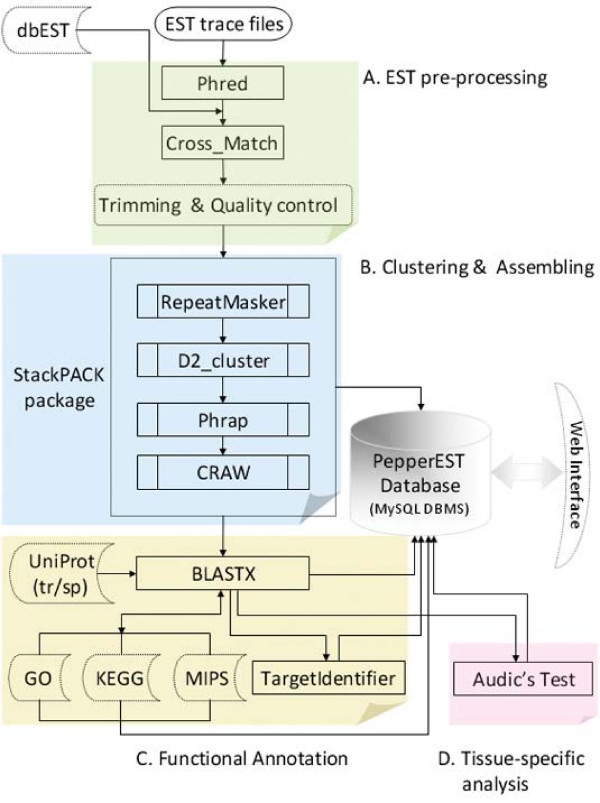
Workflow of Pepper EST database analysis.

We constructed the Pepper EST database using open source technologies, including Python (V2.4.3) scripts, MySQL (V4.0.25) database management, and PHP (5.0.5) for communicating with the database. The pipeline workflow is depicted in Figure 1.

### Dataset

The current release includes 116,412 refined ESTs from 122,582 sequenced ESTs from 21 chili pepper libraries. All libraries were constructed to represent 11 different tissues, developmental stages, or conditions of stress. Messenger RNA (mRNA) for constructing the cDNA libraries was extracted from plants of a single variety (*C. annuum *cv. Bukang) grown under the same conditions, including temperatures of 25°C/18°C (day/night) and a 16 h photoperiod [15].

### Pre-processing

EST sequences may contain a variety of contaminants, which should be removed before the sequences are used. We use Phred [16] to extract high-quality regions from raw sequence data. The Cross_Match program is used to mask contaminant and vector (pBluescriptSK^-^) sequences. Python is used to construct the trimming script to remove the masked vector, linker sequences (*EcoR*I and *Xho*I), and polyA/T regions. In addition, all short ESTs (< 100 bp) are eliminated because these are considered non-informative for EST analysis.

### Clustering and assembly

After confirming EST quality and trimming the vector sequence to obtain high-quality sequence, EST sequences are assembled into contigs to reduce inherent redundancy and to build unigene sets. Only EST sequences sharing > 96% identity over a region longer than 100 nucleotides (nts) are selected and further grouped into clusters. EST sequences in a cluster are usually defined to represent the same gene; therefore, each cluster is treated as a gene index. In the process of clustering and assembly, however, one or more consensus sequences often occur in certain EST sets within a cluster. Possible explanations for multiple contigs within a cluster include (i) alternative splicing, (ii) existence of common protein domains, or (iii) paralogy. However, the reasons for this remain to be determined since genome sequence data for chili pepper are not yet available. To address this, the sequence assembly process is performed using stackPACK™ [17] for clustering and assembling EST sequences into contigs and singletons.

The STACK (Sequence Tag Alignment and Consensus Knowledgebase) [18] system is applied to pre-processed ESTs to obtain wider gene coverage, because it employs a loose and unsupervised clustering strategy to group sequences. The d2_cluster, Phrap, and CRAW programs are incorporated into stackPACK™ to cluster, assemble, and analyze EST alignments, respectively. Consensus sequences are then merged, based on clone-identification data, to obtain the best putative gene representation (Table 1). The final clustered and assembled ESTs include a total of 22,011 unigenes, composed of 11,225 consensus genes from 8,826 clusters, and 10,786 low-complexity trimmed singletons. The pepper unigenes vary in size and range from 100 nt to 3,300 nt; however, most genes (63.7%; 7,166/11,225) range in size from 500 bp to 900 bp. The average length of consensus sequences is 1,688 bp.

**Table 1:** Parameters of the Pepper EST database

**Parameters of Pepper EST clusters and contigs**	**Number**
Pepper raw ESTs	122,582
Pepper ESTs refined	116,412
Libraries	21
Gene indices (clusters)	8,926
Transcript indices	22,810
Consensus sequences	11,225
Singletons	11,585
Full-length cDNAs clues (with E-value ≤ 1e-10)	5,685

### Annotation

Functional annotation is performed using BLAST. The filtered ESTs and assembled EST contigs are compared with the UniProt databases containing all plant protein data using the BLASTX program, with E-value set at less than 1e-3. For successful matches, only the top five hits and their alignment results are stored, annotated, and reported in the Pepper EST database. When the subject accession number is matched to the Gene Ontology (GO) [19] database, the corresponding classification is included to provide additional information on the putative functionalities. The subject accession number given to an Enzyme Commission (EC) number is also mapped onto known KEGG metabolic pathways. A total of 5,685 putative full-length cDNA clues are identified in our chili pepper EST-derived contigs and singleton data. The start codon and protein coding region are indicated in the "CDS candidate" feature on the website. We use the TargetIdentifier algorithm [20], which does not require "training" with previously known sequences and uses only the BLASTX output.

### *In silico *analysis for identification of tissue-specific genes

Additional sequence annotation is statistically analyzed to identify the tissue-specific genes among the 11,225 consensus sequences. If a particular gene has a large number of ESTs derived from a specific tissue, we assume that the gene is likely to be expressed at higher levels in that tissue [21]. We apply Audic's test [22,23] to estimate the probability of differential expression for a gene between two pools (specific-tissue versus all other tissues) of ESTs. Given the number of ESTs involved and the total number of ESTs in both pools, the differential expression of the contig in the specific tissue is evaluated. The numbers of genes differentially expressed in a total of 11 tissues are summarized in Table 2.

**Table 2:** Tissue-specific and selective genes in the Pepper EST database

**Tissues**	**No. of specific genes**^a^	**No. of selective genes**^b^
Pathogen infected leaf	131	146
Flower	54	62
Anther	2	5
Fruit	184	191
Root	58	72
Placenta	40	57
Seed	217	237
Bark	75	86
Peduncle	152	173
Callus	60	79
Seedling	127	147

^a^No. of specific genes are the numbers of contigs, which are expressed at a significantly high level in that tissue only. ^b^No. of selective genes are the numbers of contigs, which are expressed at a significantly high level in one or more tissues.

## Discussion

Pepper EST is a chili pepper EST database for EST data management and analysis. The Pepper EST database server is composed of a web interface and a MySQL database management system. The web interface is implemented in static HTML pages and PHP scripts for querying the database to allow retrieval of unigenes based on BLASTX hits and other functional annotation results. The MySQL system is used to store the collected sequence information and the analyzed data.

**Figure 2:**
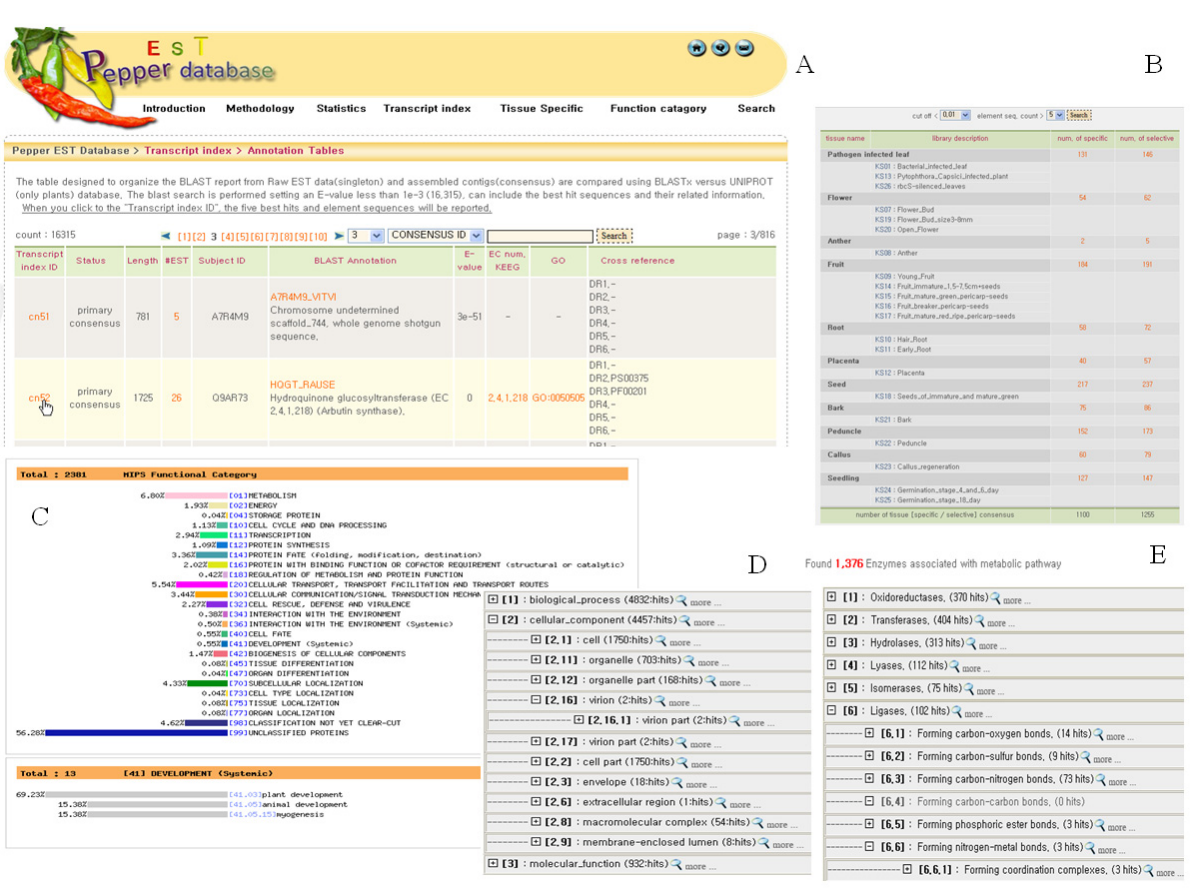
**Snapshots of the Pepper EST database web interface.** (A) Annotation tables showing the transcript index ID, #EST, and Blast Annotation of chili pepper. (B) An example showing the analysis pages for chili pepper tissue-specific genes. (C) Main menu for the MIPS functional category distribution. (D) The GO category tree menu listing all GO categories with the number of corresponding consensus sequences. (E) Example of the function pathway menu listing 1,376 chili pepper enzymes associated with metabolic pathways.

The Pepper EST database is freely accessible for querying through a web interface (Figure 2). Pepper EST can be searched and accessed via a query on the "Transcription Index" on the site. The transcription index includes the information for "Annotation Tables" and "CDS candidate." The annotation table is linked to the sequence data of 22,011 unigenes and the CDS candidate is linked to the sequence data for 5,585 full-length cDNAs. The transcription index contains annotation tables, where detailed views are available. The annotation tables show the consensus id, number of ESTs, description of ESTs, specific tissue, BLAST annotation, EC number, and GO and KEGG identifiers. The table was designed to organize the BLAST reports from EST data (singletons) and assembled contigs (one or multi-consensus), searched by BLASTX only against only the UniProt plant database. The BLASTX search was performed with E-value set at less than 1e-3 and resulted in 16,315 consensus IDs, including the best hit sequences and their related information. When users select "Transcript index ID," the five best hits and element sequences will be displayed. The consensus ID numbers ("cn") are linked to the nucleotide sequences. "#EST" links to the original sequence data from the clones in the cDNA libraries. TargetIdentifier was used to generate the CDS candidates for the identification of full-length cDNAs within a large number of EST-derived sequences. Predicting protein-coding regions from consensus EST sequences is expected to enhance the process of gene discovery and prediction of gene boundaries. EST data can be correlated with available manually-curated protein annotations, greatly enhancing the functional annotation, following six-frame translation, of ESTs.

The "Tissue-specific" page contains two tables designated "Tissue Category" and "Audic's test score". The tissue-specificity data can be analyzed by the combination of cut-off below 0.05, 0.01, or 0.001, and element sequence count of more than one, three, or five. The default tissue-specific parameters are set as a cut-off below 0.01 and an element sequence count of more than three. The tissue-specific genes analyzed by Audic's test can be specifically examined by the combinations of cut-off (below 0.05, 0.01, or 0.001), tissue name (including all, pathogen-infected leaf, flower, anther, fruit, root, placenta, seed, bark, peduncle, callus, and seedling, and), and type (specific or selective) (Table 2).

The "Functional Category" menu includes the information for the submenu for Pathway, GO, and MIPS. Pathway includes the information for 1,376 enzymes associated with metabolic pathways and includes Oxidoreductases (370 hits), Transferases (404 hits), Hydrolases (313 hits), Lyases (112 hits), Isomerases (75 hits) and Ligases (102 hits). Enzymes are divided into classes and subclasses, according to the guidelines of the Nomenclature Committee of the IUBMB. GO (Gene Ontology) contains the information regarding biological process (4,832 hits), cellular component (4,457 hits), and molecular function (932 hits). In MIPS FunCat, 2,381 genes were classified by the MIPS functional catalogue [24], which data analysis was based on the genes of *Arabidopsis*. The MIPS Functional Catalogue provides a search tool to browse the functional categories of genes with the subgroup gene function and the consensus ID and sequences.

The "Search" menu holds three different tab-style submenus for querying; these are "Annotated data", "Raw EST sequences", and "BLAST" search. The annotated data menu brings the user to a page where it is possible to search against keyword and other parameters. Raw EST sequences allow the user to search and download each pre-processed EST sequence or whole cDNA library element sequences. Users can use the BLAST search to compare their own sequences with in-house sequences in the Pepper EST database. The display of search results contains links to singleton and consensus sequences.

The unique feature of the Pepper EST database is that it is the first of its kind in that ESTs were derived from cDNA sequences from different tissues of plants of a single chili pepper variety, grown under constant growth conditions with exposure to a variety of stress agents, in one laboratory. Having incorporated the sequence data for tissue-specific expression patterns, developmental stages, and normal conditions and conditions of stress into our newly established database, we expect Pepper EST to provide a comprehensive *in silico *tool for analyzing numerous biological parameters in chili pepper plants.

## Conclusion

The Pepper EST database is a chili pepper-specific workbench for investigation of EST data. The Pepper EST database shares a subset of the same ESTs (21,000) with Pepper unigene at the Solanacea Genomics Network, however, with to the applications of significantly more ESTs and the equipment of more advanced features, the Pepper EST dabatase can provide more extensive information about chilli pepper ESTs. Pepper EST database will provide a high-quality resource for chili pepper EST analysis and also for comparative genomics for the family *Solanaceae*. In the future, the Pepper EST database will significantly aid analyses of gene synteny in the chili pepper genome and comparable studies within the family *Solanaceae*.

## Availability

The PEPPER EST database is freely available to academic researchers at  after registering on the website and obtaining approval for its use. The entire content of the database is available for download from the website. Our group will service the Pepper EST database continuously and update it annually. Questions, comments, and requests regarding this database should be sent to Dr. Cheol-Goo Hur at hurlee@kribb.re.kr

## Authors' contributions

HK and KB designed the algorithm, carried out the majority of the analyses, and prepared the manuscript. SL participated in the pre-processing of the raw EST sequences and performed the feasibility test of the tool. JK assisted in the preparation of the test data sets. BL carried out all web-programming and the construction of the database. HC and WK prepared the chili pepper EST datasets. DC conceived the study and coordinated the preparation of chili pepper EST data. CH supervised the project and participated in the design of the database. All authors read and approved the final manuscript.
